# Supplementary material for: Identification of unique cell type responses in pancreatic islets to stress
Source: Nat Commun. 2024 Jul 2;15:5567. doi: 10.1038/s41467-024-49724-w (PMC11220140; doi:10.1038/s41467-024-49724-w)
Supplement: Supplementary file 1 — Supplementary Information [file 41467_2024_49724_MOESM1_ESM.pdf]

Supplementary Information:

Identification of unique cell type responses in pancreatic islets to stress

Marlie M. Maestas[1,2], Matthew Ishahak[2], Punn Augsornworawat[3], Daniel A. Veronese-Paniagua[1,2], Kristina G. Maxwell[2,4], Leonardo Velazco-Cruz[1,2], Erica Marquez[2], Jiameng Sun[1,2], Mira Shunkarova[2], Sarah E. Gale[2], Fumihiko Urano[1,2,5], and Jeffrey R. Millman[1,2,4]*

[1]Roy and Diana Vagelos Division of Biology and Biomedical Sciences

Washington University School of Medicine, MSC 8127-057-08

660 South Euclid Avenue, St. Louis, MO 63110, USA

[2]Division of Endocrinology, Metabolism, and Lipid Research

Washington University School of Medicine, MSC 8127-057-08

[3]Department of Immunology, Faculty of Medicine Siriraj Hospital

Mahidol University, Bangkok, 10700, Thailand

[4]Department of Biomedical Engineering

Washington University in St. Louis

[5]Department of Pathology and Immunology

Washington University School of Medicine

*To whom correspondence should be addressed: Jeffrey R. Millman, jmillman@wustl.edu

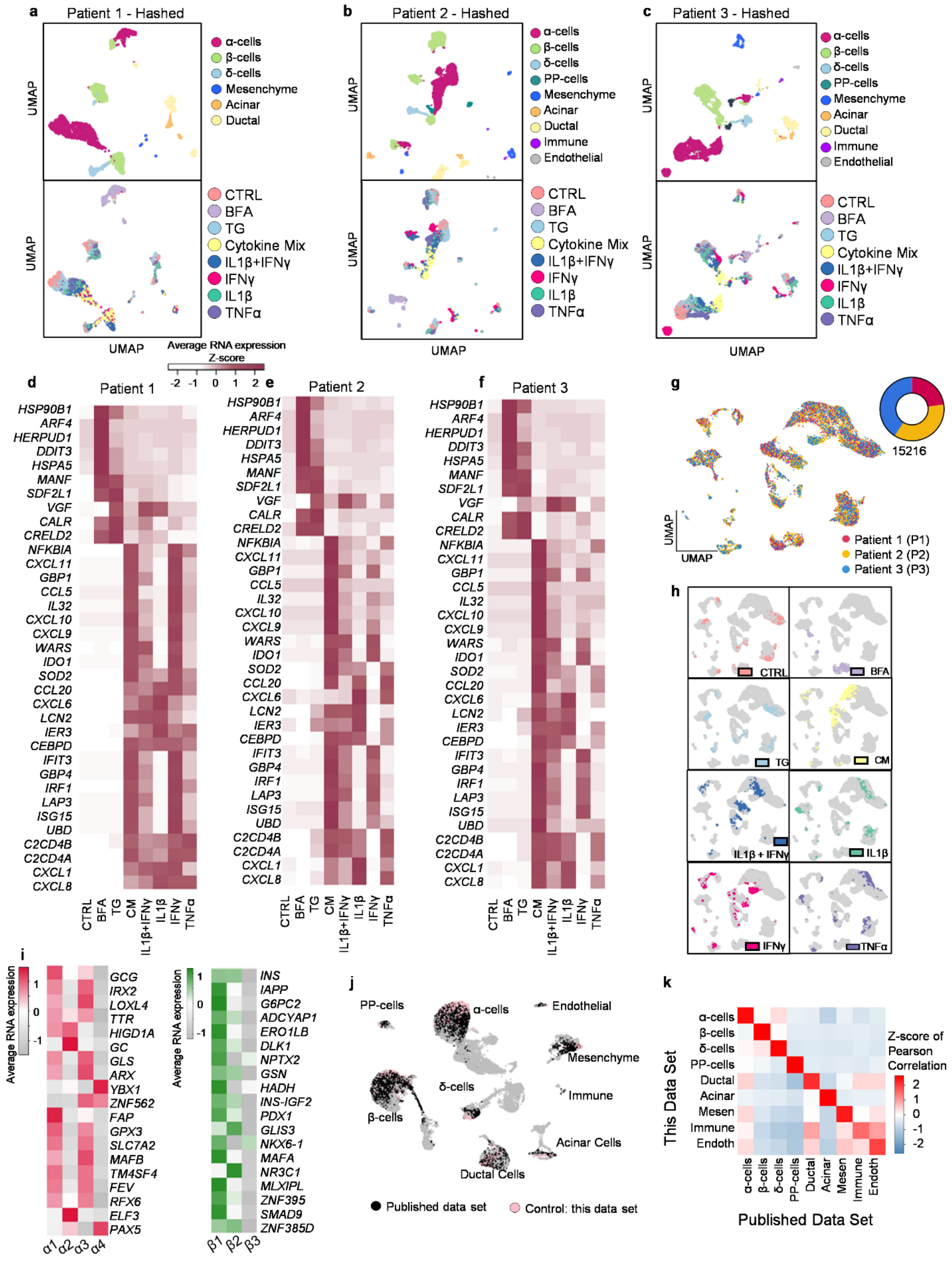

Supplementary Fig 1. Hashed single-cell RNA sequencing analysis. a-c. UMAP of cell types

(top) and conditions (bottom) **a**. Patient 1 (P1) **b.** Patient 2 (P2) **c.** Patient 3 (P3) **d.** Heatmap of shared upregulated genes (Log2 fold change> 0.25 and Adjusted P-Value of <0.05) across stressors in all cell types. **d.** P1 **e. P**2 **f.** P3. **g.** Integrated UMAP of all three hashed patients. **h.** UMAPs of each stressor within the integrated data. **i.** Heatmap comparing cell identity genes across subpopulations of α-cells (left, pink) or β-cells(right, green) **j.** Integration of published non-diabetic donor data and this data set. **k.** Pearson correlation of this data set versus the published data set. Source data are provided as a Source Data file.

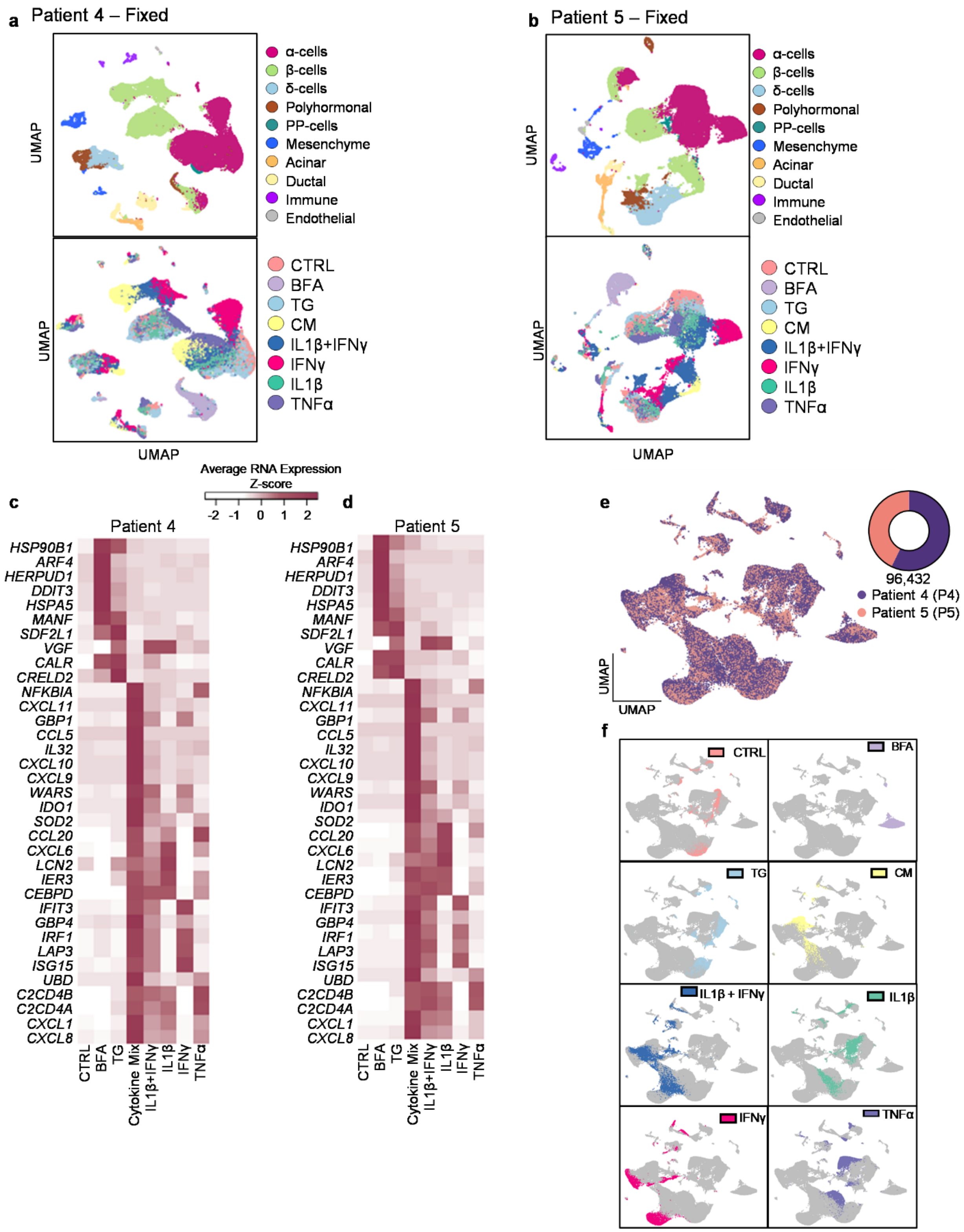

Supplementary Fig 2. Fixed single-cell RNA sequencing analysis. a-b. UMAP of cell types (top) and conditions (bottom) **a**. Patient 4 (P4) **b.** Patient 5 (P5) **c-d.** Heatmap of shared

upregulated genes (Avg. Log2 fold change> 0.25 and Adjusted P-Value of <0.05) across stressors in all cell types. **c.** P4 **d.** P5 **e.** Integrated UMAP of both fixed patients. **f.** UMAPs of each stressor within the integrated data. Source data are provided as a Source Data file.

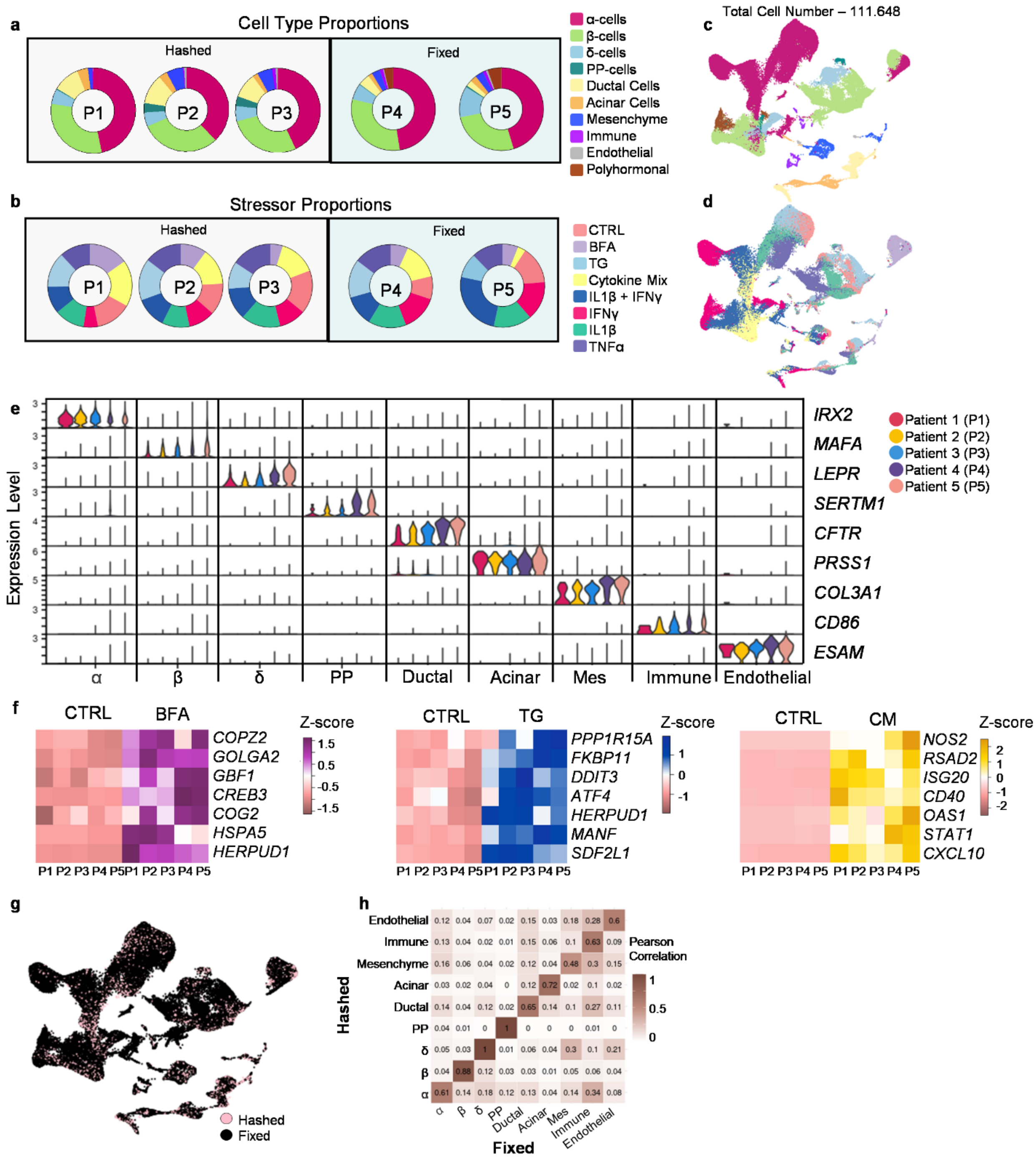

Supplementary Fig 3. Comparison of hashed versus fixed sequencing. a. Proportion of cell types in each individual patient. **b.** Proportions of stressors in each individual patient. **c.** Integrated UMAP of fixed and hashed cell types. **d.** Integrated UMAP of fixed and hashed identified by the stressors. **e.** Violin Plot of identity genes across patients and cell types, mes:

mesenchyme. **f.** Heatmap of genes found in published literature that are upregulated under stress conditions. The columns are individual patients and rows are genes, left: CTRL and BFA, middle: CTRL and TG, right: CTRL and CM. **g.** Integrated UMAP of hashed and fixed sequencing identified by sequencing type. **h.** Pearson correlation of CTRL fixed versus hashed sequencing. Source data are provided as a Source Data file.

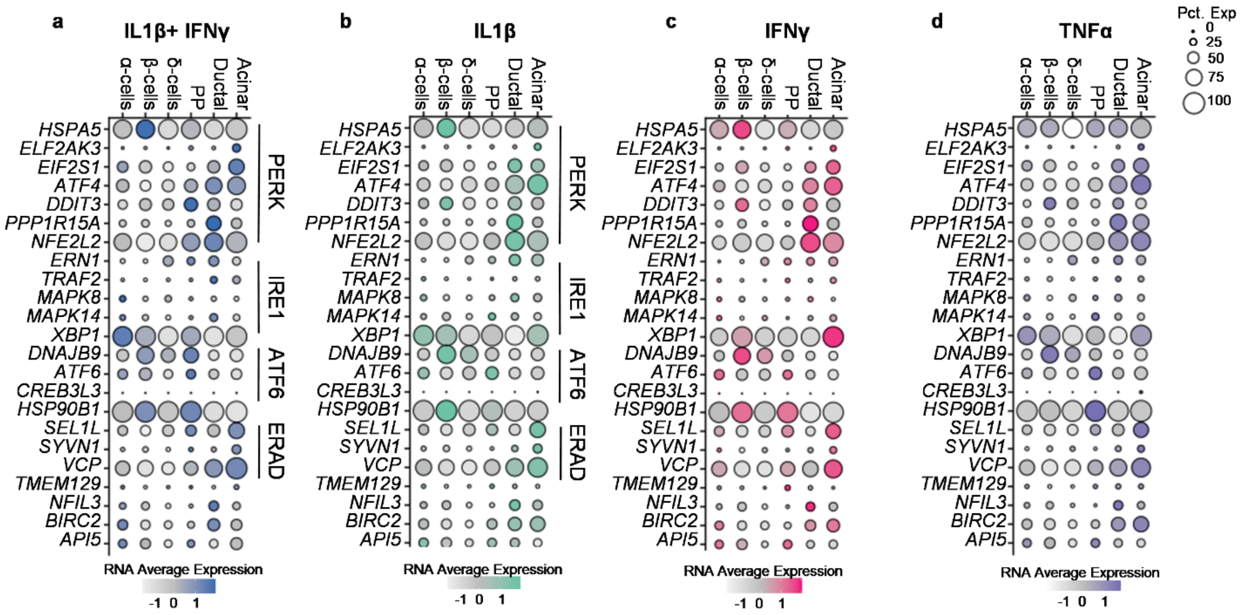

Supplementary Fig 4. Expression of UPR-associated genes in different combinations of cytokines. a, IL1β+ IFNγ dot plot of UPR and ERAD genes across cell types. **b,** IL1β dot plot of UPR and ERAD genes across cell types. **c,** IFNγ dot plot of UPR and ERAD genes across cell types. **d,** TNFα dot plot of UPR and ERAD genes across cell types.

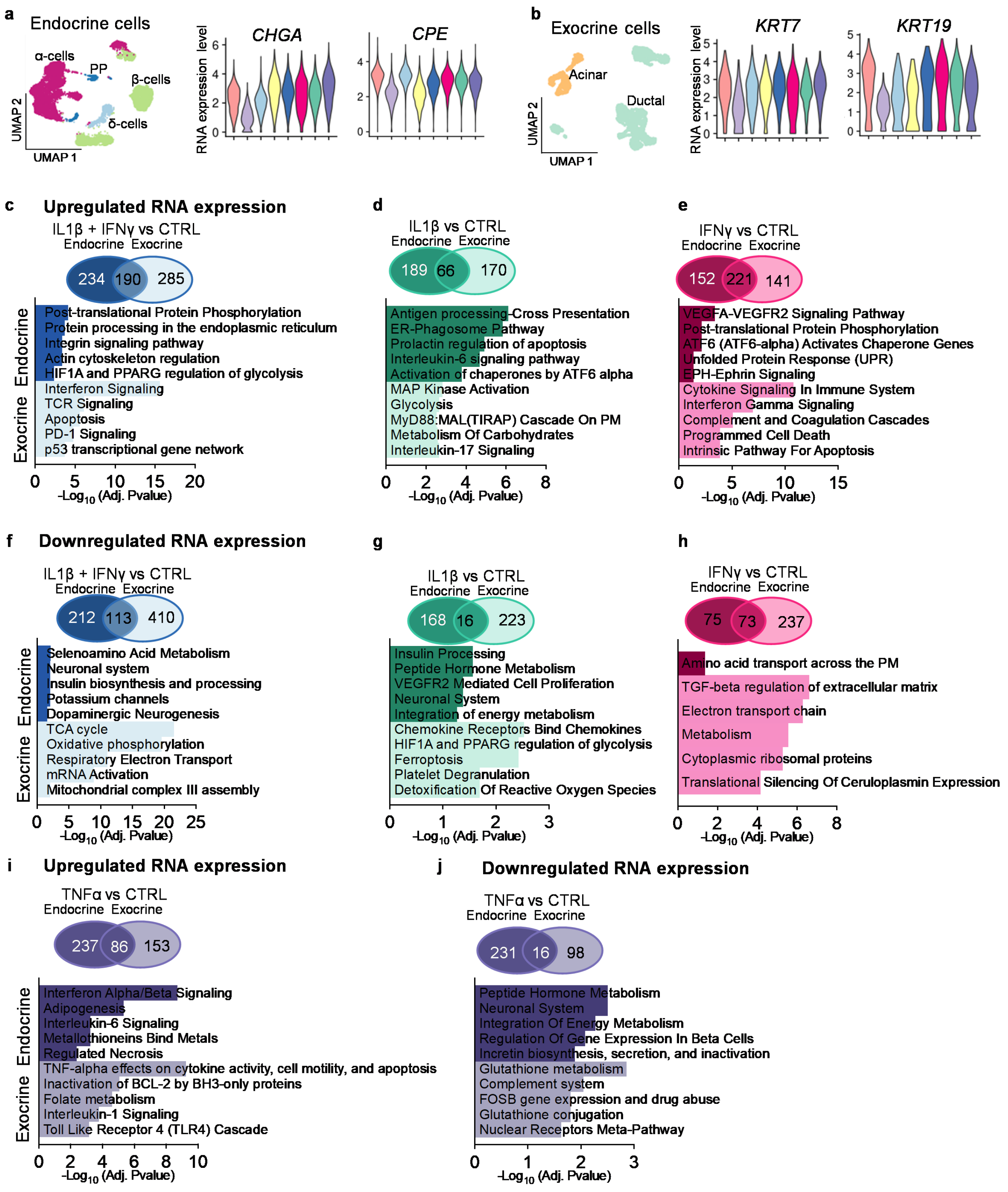

Supplementary Fig 5. Endocrine vs exocrine across cytokines. a, UMAP of re-clustered endocrine population, includes α-, β-, δ-, and PP-cells. To the right is violin plots of endocrine markers *CHGA* and *CPE*. **b,** UMAP of the re-clustered exocrine population includes ductal and

acinar cells. Violin plot of the exocrine marker, *KRT7* and *KRT19.* **c-e and i,** Venn diagram of upregulated genes (Avg $Log_2FC > 0.25$, adjusted Pvalue<0.05) stressor vs control comparing endocrine and exocrine gene lists. Bar graph using EnrichR **(c)** IL1β + IFNγ vs CTRL, **(d)** IL1β vs CTRL, **(e)** IFNγ vs CTRL, **(i)** TNFα vs CTRL. **f-h and j,** Venn diagram of downregulated genes (Avg. $Log_2FC < -0.25$, adjusted Pvalue<0.05) stressor vs control comparing endocrine and exocrine gene lists. Bar graph using EnrichR **(f)** IL1β + IFNγ vs CTRL, **(g)** IL1β vs CTRL, **(h)** IFNγ vs CTRL, **(j)** TNFα vs CTRL.

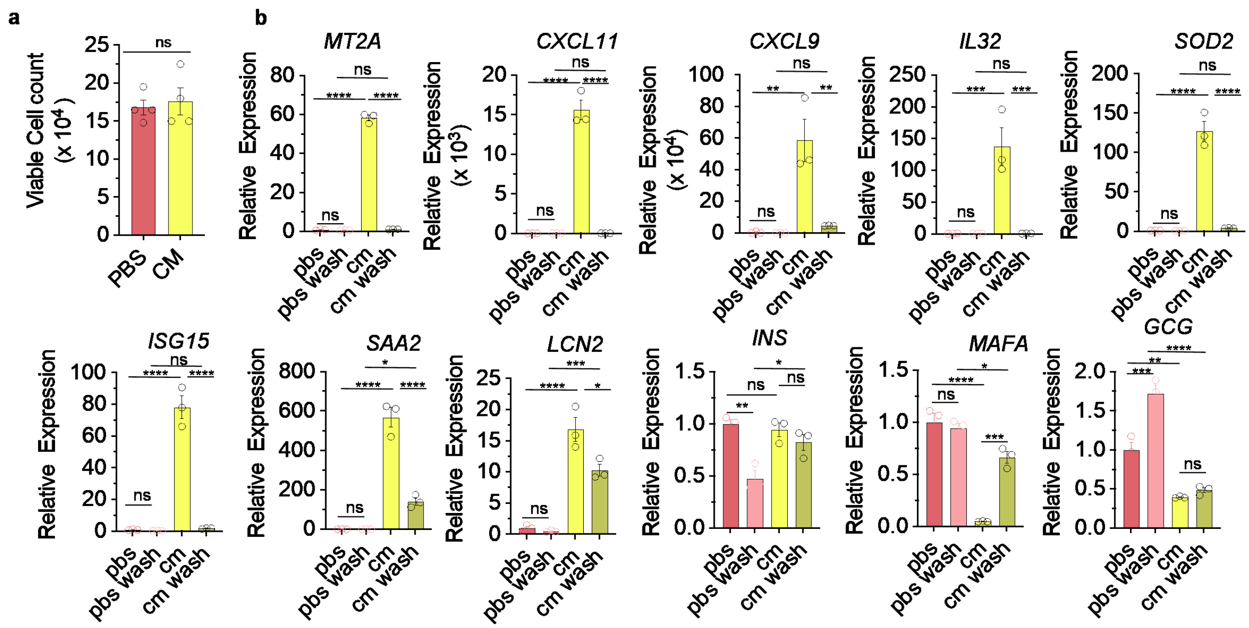

Supplementary Fig 6. CM stress in cadaveric human islets. a, Cell viability of CM treated cadaveric islets, n=4. **b,** RT-qPCR of genes associated with inflammation or islet identity. PBS : 48 hr PBS, PBS wash : 48hr PBS then 48 hr no PBS, CM : 48 hr CM, CM wash: 48 hr CM then 48 hr no CM, n=3. Unpaired t-test *P<0.05, **P<0.01,****P<0.0001, not significant (ns) and error bars represent s.e.m. Source data are provided as a Source Data file.

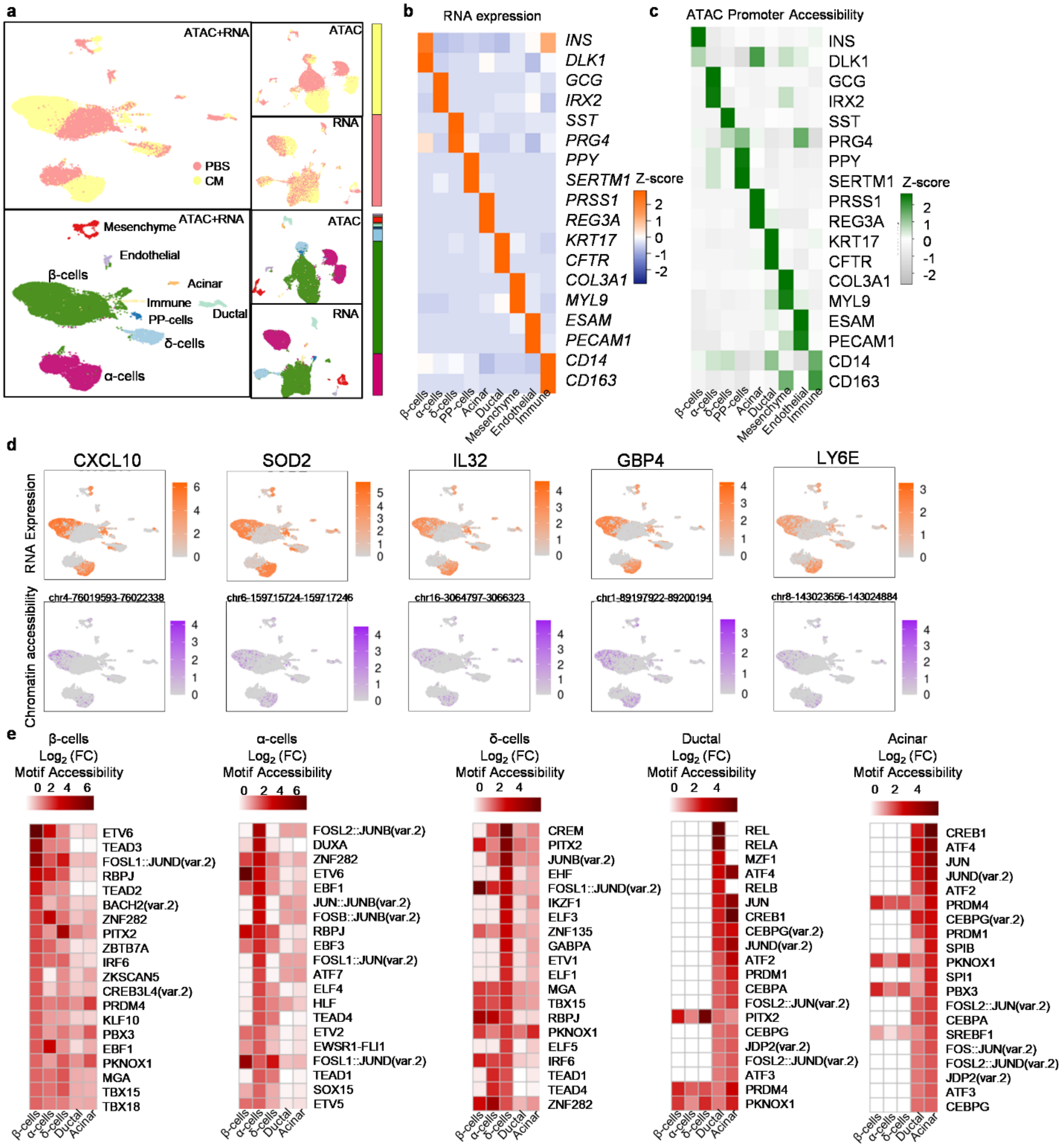

Supplementary Fig 7. snATAC of CM treated primary human islets. a, CM and PBS multi-omic snATAC sequencing, Large panel is ATAC+RNA. Top right is ATAC alone and bottom right is RNA alone. Top three are identifying which cells were treated with CM or PBS. Bottom three are identifying cell types. Bar graph is proportion of stressors or cell types. **b,** RNA

expression of canonical cell-type markers. **c,** Promoter accessibility of canonical cell-type markers. **d,** Top: UMAP of highly expressed genes under CM stress. Bottom: UMAP of corresponding chromatin accessibility. **e,** Log_2Fold Change (FC) of top 20 motif accessibility across cell types between CM and PBS. Source data are provided as a Source Data file.

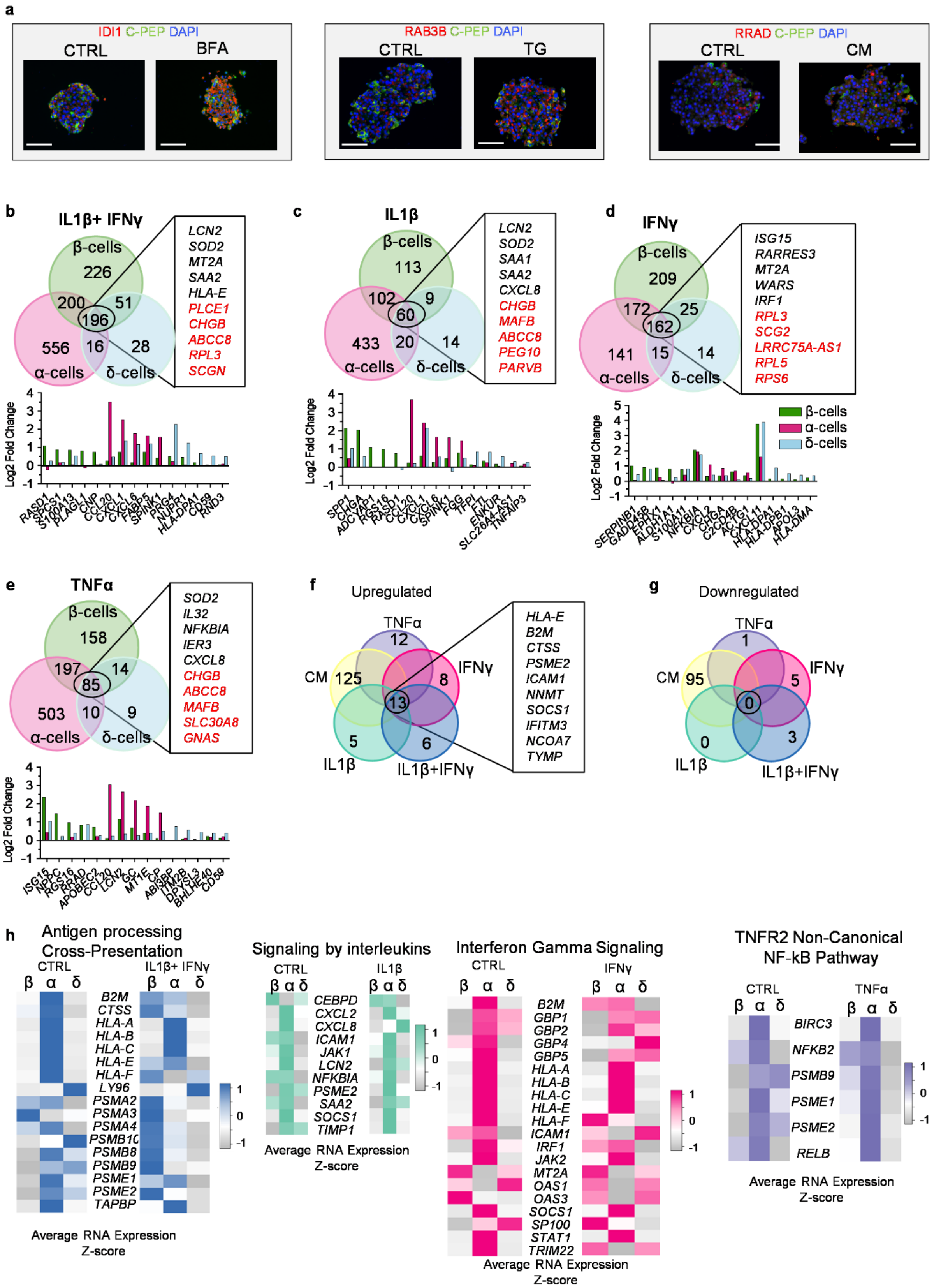

Supplementary Fig 8. Comparison of endocrine cell types under different combinations of

cytokines. a, Immunofluorescent images of primary human islets treated with BFA (right), TG (middle) or CM (left). Scale bar = 75 μm. Proteins were chosen from Fig. 4b. **b,** Venn diagram of differentially expressed genes between IL1β+ IFNγ vs control across α-, β-, and δ-cells. Below is a bar graph of Log_2 fold change of genes specific to a cell type. **c,** Venn diagram of differentially expressed genes between IL1β vs control across α-, β-, and δ-cells. Below is a bar graph of fold change of genes specific to a cell type. **d,** Venn diagram of differentially expressed genes between IFNγ vs control across α-, β-, and δ-cells. Below is a bar graph of fold change of genes specific to a cell type. **e,** Venn diagram of differentially expressed genes between TNFα vs control across α-, β-, and δ-cells. Below is a bar graph of fold change of genes specific to a cell type. **f,** Comparison of upregulated genes shared between α-, β-, and δ-cells across cytokine stressors. **g,** Comparison of downregulated genes shared between α-, β-, and δ-cells across cytokine stressors. **h,** Genes associated with Reactome signaling pathways comparing RNA expression of control vs cytokine across endocrine cell types. Source data are provided as a Source Data file.

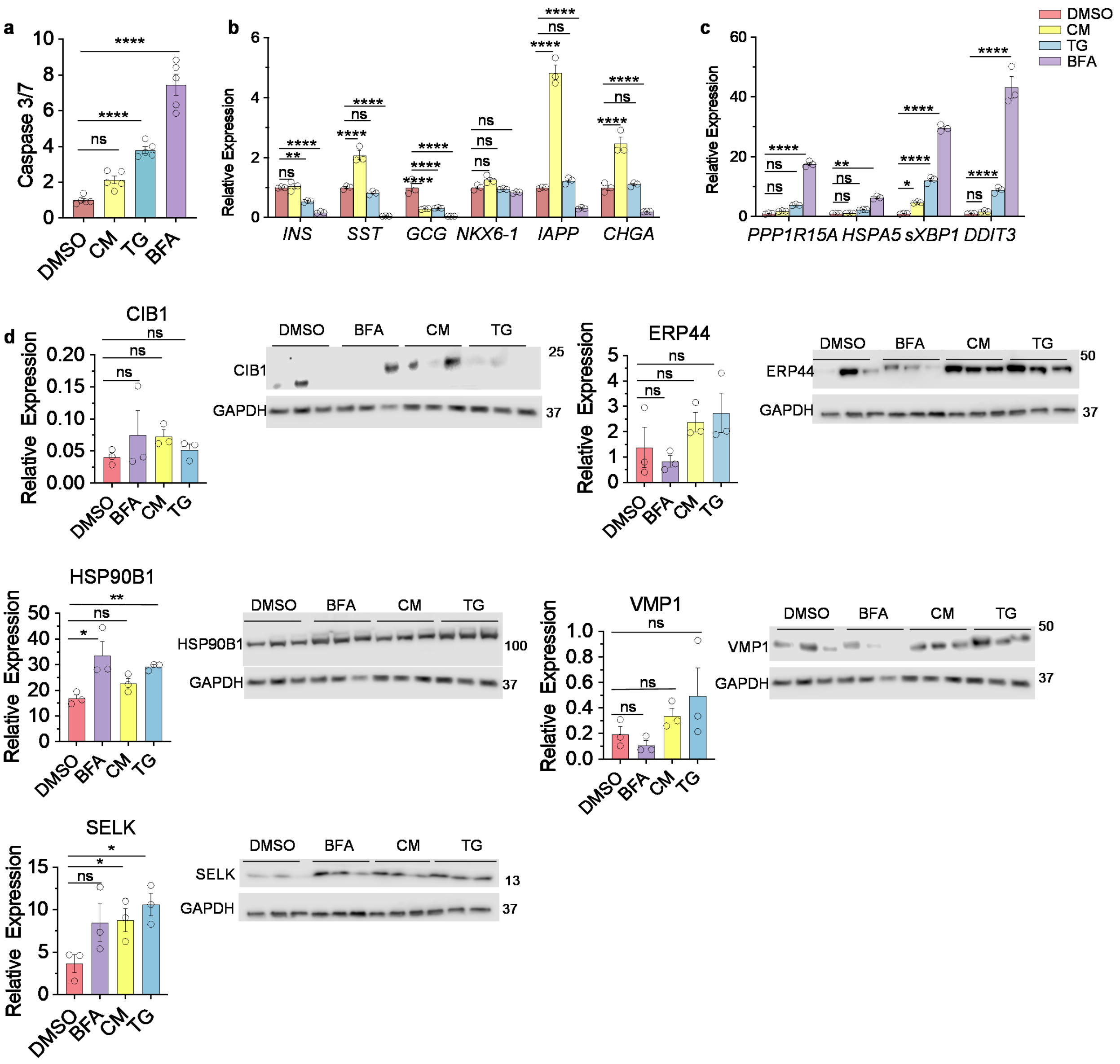

Supplementary Fig 9. SC-islet stress characterization and cadaveric islet protein expression. a, Apoptosis of SC-islets under DMSO, Cytokine Mix (CM), Thapsigargin (TG), and Brefeldin A (BFA) (n=5). **b,** RT-PCR of islet identity genes in SC-islet under different stressors (n=3). **c,** RT-PCR of UPR-associated genes in SC-islets under different stressors (n=3). **d,** Protein expression of CIB1, ERP44, HSP90B1, VMP1, and SELK. CIB1, VMP1, and HSP90B1 were run on the same blots so the GAPDH are the same for these proteins. ERP44 and SELK were run on the same blots, so the GAPDH are the same for these proteins n=3 Unpaired

t-test *P<0.05, **P<0.01,****P<0.0001, not significant (ns) and error bars represent s.e.m.

Source data are provided as a Source Data file.

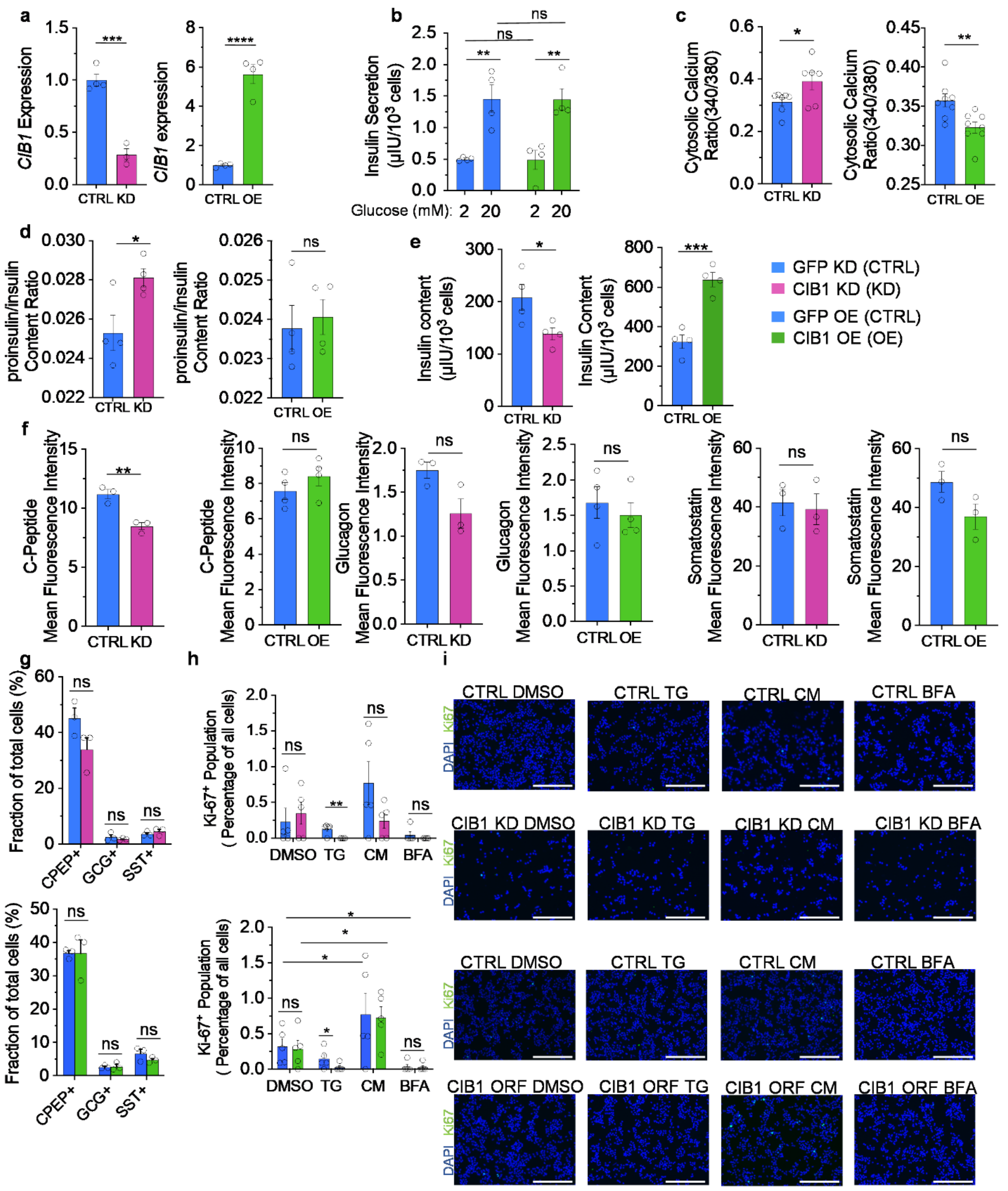

Supplementary Fig 10. CIB1 characterization in SC-islets. a, Efficiency of Knockdown using short-hairpin RNA(left) or Over Expression using open-reading-frame lentivirus (right) (N=3-4). **b,** Static Glucose stimulated insulin secretion of CTRL and *CIB1* OE (N=4). **c,** Cytosolic

calcium flux ratio of 240/280 in *CIB1* KD (left) or *CIB1* OE(right) (N=5-8). **d,** Proinsulin to insulin content ratio of *CIB1* KD (left) or *CIB1* OE (right) (N=4). **e,** Insulin content of *CIB1* KD (left) or *CIB1* OE (right) (N=4). **f,** Mean Fluorescence Intensity of C-peptide, Glucagon, and Somatostin. **g,** Fraction of cells that are Cpeptide+, Glucagon+ (GCG) or Somatostatin+ (SST) in *CIB1* KD (top) or *CIB1* OE (bottom). **h,** Ki-67+ populations as a percentage of total cells, *CIB1* KD (top) or *CIB1* OE (bottom). **i,** Fluorescent images for h, Ki-67 is in green and DAPI is blue. Scale Bar= 250 μm. Unpaired t-test, *P<0.05,**P<0.01,***P<0.001,****P<0.0001 and error bars represent s.e.m. Source data are provided as a Source Data file.

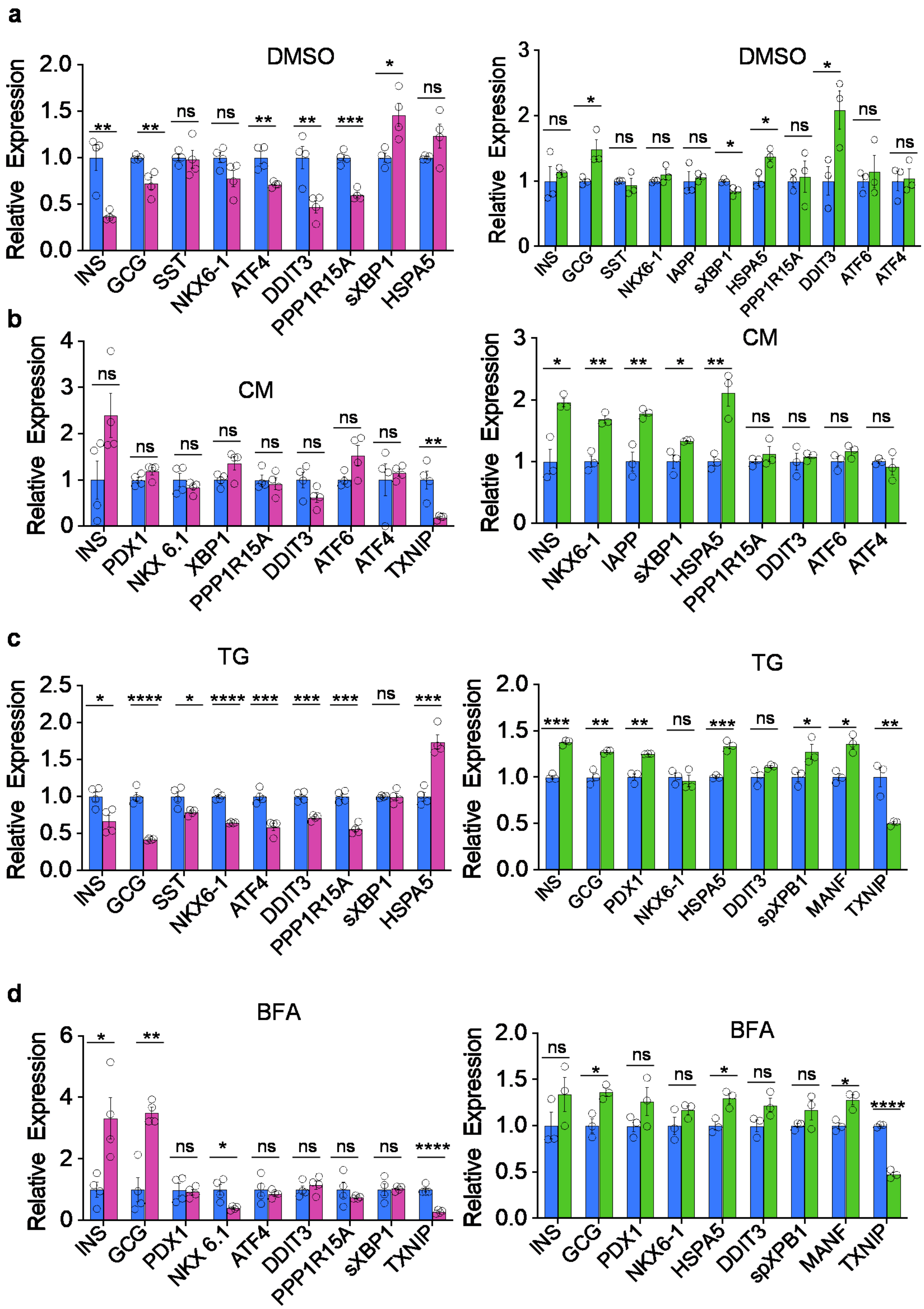

Supplementary Fig 11. qRT-PCR of *CIB1* KD or OE in SC-islets. a-d, RT-PCR, SC-islets,

CIB1 KD (right), CIB1 OE(left) **(a)** cells treated with DMSO as the control, **(b)** cells treated with CM, **(c)** TG, or **(d)** BFA. Unpaired t-test, *P<0.05,**P<0.01,***P<0.001,****P<0.0001 and error bars represent s.e.m. Source data are provided as a Source Data file.

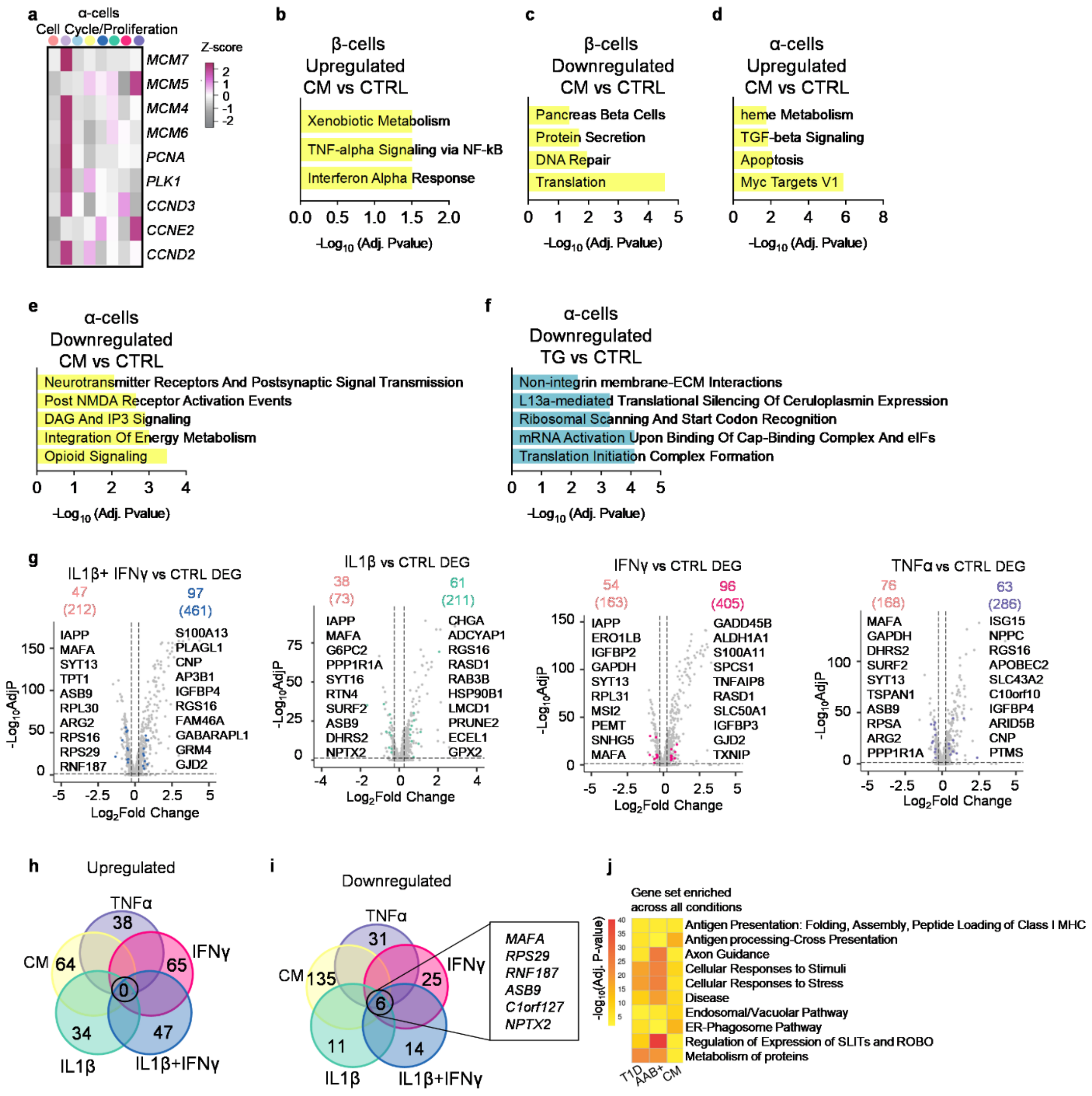

Supplementary Fig 12. 8 β- and α-cells specific stress regulation. a, Heatmap of cell cycle and proliferation-associated genes in α-cells. **b,** EnrichR pathway analysis of upregulated genes specific to β-cells under CM stress. **c,** EnrichR pathway analysis of downregulated genes specific to β-cells under CM stress. **d,** EnrichR pathway analysis of upregulated genes specific to α-cells under CM stress. **e,** EnrichR pathway analysis of downregulated genes specific to α-cells under CM stress. **f,** EnrichR pathway analysis of downregulated genes specific to α-cells under TG stress. **g,** Volcano Plot of IL1β+ IFNγ, IL1β, IFNγ, or TNFα specific DEG, the top number is specific to β-cells when compared to other cell types, the

bottom number in parenthesis is the total number of DEG. **h,** Venn diagram of upregulated genes across cytokine treatments specific to β-cells. **i,** Venn diagram of downregulated genes across cytokines specific to β-cells. **j,** Heatmap of pathways significantly upregulated in T1D, AAB+, and CM in β-cells. Source data are provided as a Source Data file.

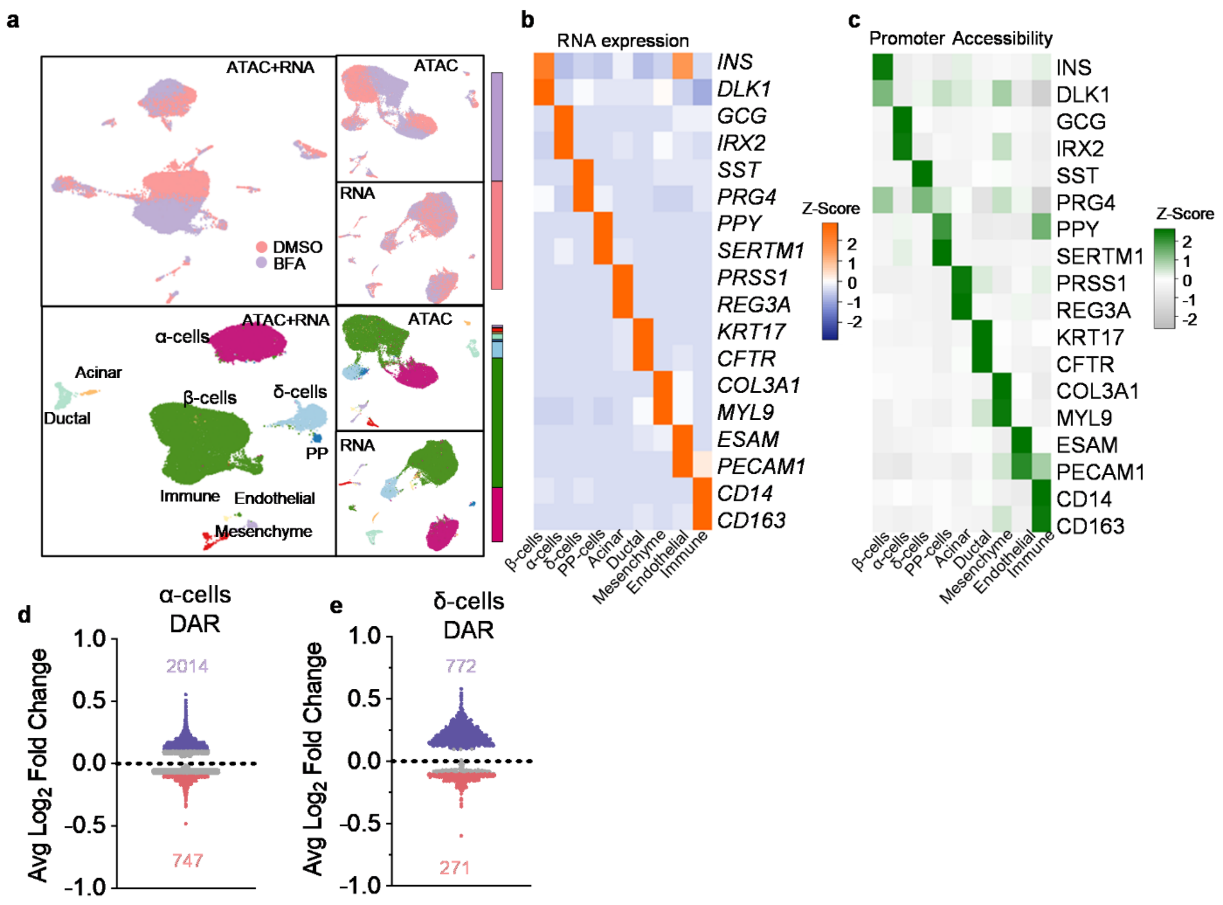

Supplementary Fig 13. snATAC from BFA treated primary human islets. a, UMAP of ATAC+RNA, ATAC alone, and RNA alone, the top three panels identify which cells were treated with BFA or DMSO, and the bottom three panels identify cell types. Bar graphs to the right are the proportion of stressors or cell types in the entire population. **b,** RNA expression of canonical markers to identify cell types. **c,** Promoter accessibility of canonical markers. **d,** Differentially accessible regions across the genome in α-cells. **e,** Differentially accessible regions in δ-cells. Source data are provided as a Source Data file.
