# Supplementary material for: Identification of unique cell type responses in pancreatic islets to stress
Source: Nat Commun. 2024 Jul 2;15:5567. doi: 10.1038/s41467-024-49724-w (PMC11220140; doi:10.1038/s41467-024-49724-w)
Supplement: Supplementary file 3 — Description of Additional Supplementary Files [file 41467_2024_49724_MOESM3_ESM.pdf]

Description of Additional Supplementary Files

Supplementary Data Legends:

Supplementary Data 1. Materials. This table is the list of reagents, cell lines, antibodies, and sequences used in this study. This includes ER and inflammatory stressors, hPSC cell line, SC-islet differentiation protocol, base medium formulation, factors and compounds for SC-islet differentiation, qPCR primer sequences, CIB1 sequence, drug compounds, and protein antibodies.

Supplementary Data 2. Differentially expressed genes of patient 1. These are tables for differentially expressed genes of patient 1 between BFA vs CTRL, TG vs CTRL, CM vs CTRL, IFNG vs CTRL, IL1B vs CTRL, IL1BIFNG vs CTRL, and TNFA vs CTRL in α-, β-, δ-, acinar, or ductal cells. The threshold is p_val_adj <0.05, and absolute Avg_log2FC >|0.25|. Statistical significance was determined by a two-sided Wilcoxon rank sum test.

Supplementary Data 3. Differentially expressed genes of patient 2. These are tables for differentially expressed genes of patient 2 between BFA vs CTRL, TG vs CTRL, CM vs CTRL, IFNG vs CTRL, IL1B vs CTRL, IL1BIFNG vs CTRL, and TNFA vs CTRL in α-, β-, δ-, pp, acinar, or ductal cells. The threshold is p_val_adj <0.05, and absolute Avg_log2FC >|0.25|. Statistical significance was determined by a two-sided Wilcoxon rank sum test.

Supplementary Data 4. Differentially expressed genes of patient 3. These are tables for differentially expressed genes of patient 3 between BFA vs CTRL, TG vs CTRL, CM vs CTRL, IFNG vs CTRL, IL1B vs CTRL, IL1BIFNG vs CTRL, and TNFA vs CTRL in α-, β-, δ-, pp, acinar, or ductal cells. The threshold is p_val_adj <0.05, and absolute Avg_log2FC >|0.25|. Statistical significance was determined by a two-sided Wilcoxon rank sum test.

Supplementary Data 5. Differentially expressed genes of patient 4. The first table shows the sequences and probes used on patient 4 for fixed single-cell RNA sequencing. The rest of the tables are for differentially expressed genes of patient 4 between BFA vs CTRL, TG vs CTRL, CM vs CTRL, IFNG vs CTRL, IL1B vs CTRL, IL1BIFNG vs CTRL, and TNFA vs CTRL in α-, β-, δ-, pp, poly hormonal, acinar, or ductal cells. The threshold is p_val_adj <0.05, and absolute Avg_log2FC >|0.25|. Statistical significance was determined by a two-sided Wilcoxon rank sum test.

Supplementary Data 6. Differentially expressed genes of patient 5. The first table shows the sequences and probes used on patient 5 for fixed single-cell RNA sequencing. The rest of the tables are for differentially expressed genes of patient 5 between BFA vs CTRL, TG vs CTRL, CM vs CTRL, IFNG vs CTRL, IL1B vs CTRL, IL1BIFNG vs CTRL, and TNFA vs CTRL in α-, β-, δ-, pp, poly hormonal, acinar, or ductal cells. The threshold is p_val_adj <0.05, and absolute Avg_log2FC >|0.25|. Statistical significance was determined by a two-sided Wilcoxon rank sum test.

Supplementary Data 7. Differentially expressed genes in alpha subpopulations and beta subpopulations. (7.1) is α-1 population vs α-2, α-3, and α-4 (7.2) is α-2 population vs α-1, α-3, and α-4 (7.3) is α-3 vs α-1, α-2, and α-4 (7.4) is α-4 vs α-1, α-2, α-3 (7.5) β-1 population vs β-2 and β-3 (7.6) β-2 vs β-1 and β-3 (7.7) β-3 vs β-1 and β-2. The threshold is p_val_adj <0.05, and absolute Avg_log2FC >|0.25|. Statistical significance was determined by a two-sided Wilcoxon rank sum test.

Supplementary Data 8. Differentially expressed genes in endocrine cells and exocrine cells. This table has differentially expressed genes in endocrine cells (α-, β-, δ-, and pp) BFA vs CTRL, TG vs CTRL, CM vs CTRL, IFNG vs CTRL, IL1B vs CTRL, IL1BIFNG vs CTRL, and TNFA vs CTRL. This table also has differentially expressed genes in exocrine cells (acinar and ductal cells) BFA vs CTRL, TG vs CTRL, CM vs CTRL, IFNG vs CTRL, IL1B vs CTRL, IL1BIFNG vs CTRL, and TNFA vs CTRL. The threshold is p_val_adj <0.05, and absolute Avg_log2FC >|0.25|. Statistical significance was determined by a two-sided Wilcoxon rank sum test. This table also includes motif chromatin accessibility of exocrine and endocrine tissue. Statistical significance assessed by two-sided logistic regression for motif chromatin accessibility.

Supplementary Data 9. EnrichR pathway analysis in endocrine and exocrine tissue. These tables are the endocrine and exocrine pathway analyses for the differentially expressed genes from Table 8. EnrichR uses the Benjamini-Hochberg method to correct for multiple hypotheses testing.

Supplementary Data 10. Differentially expressed genes from integration in α-, β-, and δ-cells. These tables are from subsetting cell types α-, β-, and δ-cells separately and then comparing stressors to the control. BFA vs CTRL, TG vs CTRL, CM vs CTRL, IFNG vs CTRL, IL1B vs CTRL, IL1BIFNG vs CTRL, and TNFA vs CTRL in α-, β-, and δ-cells. The threshold is p_val_adj <0.05, and absolute Avg_log2FC >|0.25|. Statistical significance was determined by a two-sided Wilcoxon rank sum test.

Supplementary Data 11. EnrichR pathway analysis in shared and specific pathways under BFA and CM stress. These tables are the pathway analysis for the differentially expressed genes from Table 10. EnrichR uses the Benjamini-Hochberg method to correct for multiple hypotheses testing.

Supplementary Data 12. CIB1 fold change under stress. This table shows the avg_log2FC of BFA vs CTRL, TG vs CTRL, CM vs CTRL, IFNG vs CTRL, IL1B vs CTRL, IL1BIFNG vs CTRL, and TNFA vs CTRL of CIB1 in α-, β-, δ-, pp, acinar, and ductal cells. Statistical significance was determined by a two-sided Wilcoxon rank sum test.

Supplementary Data 13. Genes that are specifically regulated in only β-cells. This table shows the differentially expressed genes specific to β-cells comparing BFA vs CTRL, TG vs CTRL, CM vs CTRL, IFNG vs CTRL, IL1B vs CTRL, IL1BIFNG vs CTRL, and TNFA vs CTRL. The threshold is p_val_adj <0.05, and absolute Avg_log2FC >|0.25|. Statistical significance was determined by a two-sided Wilcoxon rank sum test.

Supplementary Data 14. EnrichR pathway analysis of β-cell specific genes. This table shows the pathways associated with the genes found in Table 12. EnrichR uses the Benjamini-Hochberg method to correct for multiple hypotheses testing.

Supplementary Data 15. Genes that are specifically regulated in only α-cells. This table shows the differentially expressed genes specific to α-cells comparing BFA vs CTRL, TG vs CTRL, CM vs CTRL, IFNG vs CTRL, IL1B vs CTRL, IL1BIFNG vs CTRL, and TNFA vs CTRL. The threshold is p_val_adj <0.05, and absolute Avg_log2FC >|0.25|. Statistical significance was determined by a two-sided Wilcoxon rank sum test.

Supplementary Data 16. EnrichR pathway analysis of α-cells specific genes. This table shows the pathways associated with the genes found in Table 14. EnrichR uses the Benjamini-Hochberg method to correct for multiple hypotheses testing.

Supplementary Data 17. Differentially expressed genes in T1D and AAB+. These tables show the differentially expressed genes in T1D vs CTRL and AAB+ vs CTRL in β-cells. The threshold is p_val_adj

<0.05, and absolute Avg_log2FC >|0.25|. This also includes the EnrichR analysis of upregulated genes. Statistical significance was determined by a two-sided Wilcoxon rank sum test.

Supplementary Data 18. Differentially accessible regions in β-, α-, and δ-cells. The first three tables are differentially accessible regions BFA vs CTRL in β-, α-, or δ-cells. This table also includes the Log2FC of overlapping upregulated genes and upregulated motifs in β-cells. For gene expression statistical significance was determined by a two-sided Wilcoxon rank sum test for motif enrichment Benjamini-Hochberg method to correct for multiple hypotheses testing.
