# Supplementary material for: Identification of unique cell type responses in pancreatic islets to stress
Source: Nat Commun. 2024 Jul 2;15:5567. doi: 10.1038/s41467-024-49724-w (PMC11220140; doi:10.1038/s41467-024-49724-w)
Supplement: Supplementary file 5 — Reporting Summary [file 41467_2024_49724_MOESM5_ESM.pdf]

nature portfolio

Corresponding author(s): Jeffrey R. Millman

Last updated by author(s): May 7, 2024

Reporting Summary

Nature Portfolio wishes to improve the reproducibility of the work that we publish. This form provides structure for consistency and transparency in reporting. For further information on Nature Portfolio policies, see our Editorial Policies and the Editorial Policy Checklist.

Statistics

For all statistical analyses, confirm that the following items are present in the figure legend, table legend, main text, or Methods section.

n/a	Confirmed	
☐	☒	The exact sample size (*n*) for each experimental group/condition, given as a discrete number and unit of measurement
☐	☒	A statement on whether measurements were taken from distinct samples or whether the same sample was measured repeatedly
☐	☒	The statistical test(s) used AND whether they are one- or two-sided<br>*Only common tests should be described solely by name; describe more complex techniques in the Methods section.*
☒	☐	A description of all covariates tested
☐	☒	A description of any assumptions or corrections, such as tests of normality and adjustment for multiple comparisons
☐	☒	A full description of the statistical parameters including central tendency (e.g. means) or other basic estimates (e.g. regression coefficient) AND variation (e.g. standard deviation) or associated estimates of uncertainty (e.g. confidence intervals)
☐	☒	For null hypothesis testing, the test statistic (e.g. *F*, *t*, *r*) with confidence intervals, effect sizes, degrees of freedom and *P* value noted<br>*Give P values as exact values whenever suitable.*
☒	☐	For Bayesian analysis, information on the choice of priors and Markov chain Monte Carlo settings
☒	☐	For hierarchical and complex designs, identification of the appropriate level for tests and full reporting of outcomes
☐	☒	Estimates of effect sizes (e.g. Cohen's *d*, Pearson's *r*), indicating how they were calculated

Our web collection on statistics for biologists contains articles on many of the points above.

Software and code

Policy information about availability of computer code

Data collection	Single cell sequencing data was collected using the 10x chromium and illumina NovaSeq 6000. Flow cytometry data was collected using cytek software and Flowjo. qPCR data was collected from the QuantStudio6 using Design & Analysis 2.6.0. Fluorescent images were collected from Leica DMi.
Data analysis	Single-cell RNA sequencing data was analyzed R version 4.0.3. We used packages Seurat v4.3.0, EnhancedVolcano v1.8.0, gplots v3.1.3, SCENIC v1.3.1, EnrichR interactive website, Signac v1.3.0 ,chromVar v1.12.0, JASPAR2020 v0.99.10, ggplot2 v3.4.0, dplyr v1.0.10, devtools v2.3.2, GenomicRanges v1.26.0, chromVar 1.12.0.

For manuscripts utilizing custom algorithms or software that are central to the research but not yet described in published literature, software must be made available to editors and reviewers. We strongly encourage code deposition in a community repository (e.g. GitHub). See the Nature Portfolio guidelines for submitting code & software for further information.

Data

Policy information about availability of data

All manuscripts must include a data availability statement. This statement should provide the following information, where applicable:

- Accession codes, unique identifiers, or web links for publicly available datasets
- A description of any restrictions on data availability
- For clinical datasets or third party data, please ensure that the statement adheres to our policy

All scRNA-seq and ATAC-seq analysis data can be found in Supplementary Data. Sequencing data have been deposited to gene expression omnibus (GEO) under accession code GSE237448. Source data are provided with this paper.

Research involving human participants, their data, or biological material

Policy information about studies with human participants or human data. See also policy information about sex, gender (identity/presentation), and sexual orientation and race, ethnicity and racism.

Reporting on sex and gender	6 males and 2 females were used in this data set
Reporting on race, ethnicity, or other socially relevant groupings	1 American Indian, 1 Hawaiian, 2 Caucasian, and 4 Hispanic people were used in this study.
Population characteristics	Donors for human adult islets ranged from 22.7-33.3 BMI, 26-65 years of age, and HbA1c 5.2%-5.9%
Recruitment	Prodo labs was apart of the recruitment of donors. We did not perform recruitment due to data being assembled from published sources.
Ethics oversight	Our research complies with all relevant ethical regulations. The research in this study was approved by the Washington University Institutional Biological & Chemical (IBC) Safety Committee (Approval number 12186). Washington University Embryonic Stem Cell Research Oversight Committee approved all work utilizing HUES8 (Approval number 15-002).

Note that full information on the approval of the study protocol must also be provided in the manuscript.

Field-specific reporting

Please select the one below that is the best fit for your research. If you are not sure, read the appropriate sections before making your selection.

☒ Life sciences ☐ Behavioural & social sciences ☐ Ecological, evolutionary & environmental sciences

For a reference copy of the document with all sections, see nature.com/documents/nr-reporting-summary-flat.pdf

Life sciences study design

All studies must disclose on these points even when the disclosure is negative.

Sample size	Sample sizes for sequencing determined based on 10x Genomics recommended instructions. Target sequencing cell number was 10000. Sample sizes in other experiments were based on previous experiences and studies. No statistical analysis was performed to determine sample size but were similar to other previous publications from our lab (Hogrebe et al., Augsornworawat et al.)
Data exclusions	Poor quality cells including dead cells, doublets and poorly sequenced cells were excluded from this study. In general, poor quality and dead cells were defined as those with greater than 25% of reads mapping to the mitochondrial genome. Doublets and poorly sequenced cells were filtered based on low number of gene counts and/or aberrantly high gene counts. Specific filtering thresholds are included in Supplementary Tables.
Replication	Human islet sequencing was obtained from five different non-diabetic donors and were compared to each other.
Randomization	Randomization is not applicable in this study because there are no human participants and no clinical trials.
Blinding	Data collection and analysis were not performed blind to the conditions of the experiments. Blinding was not used in this study because there are no human participants that is subject to bias.

Reporting for specific materials, systems and methods

We require information from authors about some types of materials, experimental systems and methods used in many studies. Here, indicate whether each material, system or method listed is relevant to your study. If you are not sure if a list item applies to your research, read the appropriate section before selecting a response.

Materials & experimental systems

n/a	Involved in the study
[ ]	[x] Antibodies
[ ]	[x] Eukaryotic cell lines
[x]	[ ] Palaeontology and archaeology
[x]	[ ] Animals and other organisms
[x]	[ ] Clinical data
[x]	[ ] Dual use research of concern
[x]	[ ] Plants

Methods

n/a	Involved in the study
[x]	[ ] ChIP-seq
[ ]	[x] Flow cytometry
[x]	[ ] MRI-based neuroimaging

Antibodies

Antibodies used

CIB1 (Protein Tech, 67901-1-Ig, 1:500), ERp44 (Cell signaling, 2886, 1:500), HSP90B1 (R&D Systems, MAB7606, 1:2500), VMP1 (Cell Signaling, 12929, 1:250), SELK (Abcam, AB121276, 1:250), GAPDH (Santa Cruz, sc-32233, 1:1000), IDI1 (Abcam, ab242049, 1:400), RAB3B (proteintech, 15774-1-AP, 1:200), RRAD (Abcam, ab177151, 1:400), Ki67(Abcam, ab16667, 1:300), C-peptide (DSHB, GN-ID4-S, 1:300) SST (BD bioscience, 566032, 1:250), GCG (BD bioscience, 2318824, 1:350)

Secondary antibodies: anti-rate alexa fluor 488 (invitrogen, A21208, 1:300), anti-rabbit alexa fluor 488 (invitrogen, a21206, 1:300), anti-mouse alexa flour 594 (invitrogen, a21203, 1:300), anti0rabbit alexa fluor 647 (invitrogen, A31573, 1:300), Donkey anti-mouse (Jackson immunoResearch, 715-005-151, 1:5000), donkey anti-rabbit (Jackson ImmunoResearch, 711035152, 1:5000), Anti-rat PE (Jackson ImmunoResearch, 712-116-153, 1:300)

Validation

All antibodies used have been validated on the supplier web page.

Eukaryotic cell lines

Policy information about cell lines and Sex and Gender in Research

Cell line source(s)

HUES8 a hESC line provided by Douglas Melton (Harvard University)
Lenti-X 293T cell line (Takara; 632180)

Authentication

All lines have been authenticated with DNA fingerprinting.

Mycoplasma contamination

Hues8 tested negative for mycoplasma by Washington University GEiC.

Commonly misidentified lines
(See ICLAC register)

No cell lines listed used

Plants

Seed stocks

N/A

Novel plant genotypes

N/A

Authentication

N/A

Flow Cytometry

Plots

Confirm that:

- [ ] The axis labels state the marker and fluorochrome used (e.g. CD4-FITC).
- [x] The axis scales are clearly visible. Include numbers along axes only for bottom left plot of group (a 'group' is an analysis of identical markers).
- [x] All plots are contour plots with outliers or pseudocolor plots.
- [x] A numerical value for number of cells or percentage (with statistics) is provided.

Methodology

Sample preparation	The cells were fixed with 4% paraformaldehyde aqueous solution (PFA, 157-4-100) for 20 min at RT. Then PFA was removed, and cells were washed with PBS. Blocking and permeabilized were done using 5% donkey serum and 0.1% Triton-X in PBS for 30 min on ice. Cells were then incubated in primary antibody overnight at 4°C. Next day secondary was added and incubated for 2 hr in the dark.
Instrument	Cytek Northern Lights
Software	FlowJo v10.8.1 was used for analysis
Cell population abundance	N/A, we do not have post-sort fractions in this study.
Gating strategy	Gating strategy was based off of a negative control. An example can be found in Source Data Fig. 10

☒ Tick this box to confirm that a figure exemplifying the gating strategy is provided in the Supplementary Information.
